# Identification of Antimicrobial-Resistant Zoonotic Bacteria in Swine Production: Implications from the One Health Perspective

**DOI:** 10.3390/antibiotics13090883

**Published:** 2024-09-13

**Authors:** Maria Paz Ventero, Clara Marin, Lourdes Migura-Garcia, Carla Tort-Miro, Noemi Giler, Inmaculada Gomez, Isabel Escribano, Ana Marco-Fuertes, Laura Montoro-Dasi, Laura Lorenzo-Rebenaque, Santiago Vega, Maria Teresa Pérez-Gracia, Juan Carlos Rodríguez

**Affiliations:** 1 Servicio de Microbiología, Hospital General Universitario Dr. Balmis, Instituto de Investigación Sanitaria y Biomédica de Alicante (ISABIAL), 03010 Alicante, Spain; ventero_mar@isabial.es (M.P.V.); inmagg1998@gmail.com (I.G.); escribano_isacan@gva.es (I.E.); rodriguez_juadia@gva.es (J.C.R.); 2 Facultad de Veterinaria, Instituto de Ciencias Biomédicas, Universidad Cardenal Herrera—CEU, CEU Universities, Alfara del Patriarca, 46115 Valencia, Spain; ana.marco3@alumnos.uchceu.es (A.M.-F.); laura.montoro@uchceu.es (L.M.-D.); svega@uchceu.es (S.V.); 3 Unitat Mixta d’Investigació IRTA-UAB en Sanitat Animal, Centre de Recerca en Sanitat Animal (CReSA), Campus de la Universitat Autònoma de Barcelona, 08193 Bellaterra, Spain; carla.tort@irta.cat (C.T.-M.); noemi.giler@irta.cat (N.G.); 4 IRTA. Programa de Sanitat Animal, CReSA, Collaborating Centre of the World Organisation for Animal Health for Research and Control of Emerging and Re-Emerging Pig Diseases, Europe Campus de la UAB, 08193 Bellaterra, Spain; 5 Institute of Science and Animal Technology, Universitat Politècnica de Valencia, 46022 Valencia, Spain; laulore@upv.es; 6 Área de Microbiología, Departamento de Farmacia, Instituto de Ciencias Biomédicas, Facultad de Ciencias de la Salud, Universidad Cardenal Herrera–CEU, CEU Universities, Alfara del Patriarca, 46115 Valencia, Spain; teresa@uchceu.es; 7 Departamento de Producción Vegetal y Microbiología, Universidad Miguel Hernández de Elche, 03010 Alicante, Spain

**Keywords:** global health, pig farms, multidrug resistant microorganism

## Abstract

Antimicrobial resistance poses a major threat to global health and food security and is primarily driven by antimicrobial use in human and veterinary medicine. Understanding its epidemiology at farm level is crucial for effective control measures. Despite the significant reduction in antibiotic use in conventional livestock production, the swine sector traditionally has a higher level of antibiotic use in veterinary medicine. Consequently, multidrug resistance (MDR) among microbial isolates of swine origin has been relatively frequent. The aim of this study was to assess the presence of multidrug-resistant (MDR) bacteria, enteric pathogens and resistance genes to the main antibiotics used in clinical practice, both within the environment and in animals across pig farms characterized by varying degrees of sanitary status. A total of 274 samples were collected. Of these, 34 samples were collected from the environment (wall swabs, slat swabs and slurry pit), and 240 samples were collected from animals (sows’ and piglets’ rectal faeces). All samples were analysed for MDR bacteria and enteric pathogens. The study revealed a high frequency of extended-spectrum beta-lactamases (ESBL)-producing *Enterobacterales* and *Campylobacter* spp., with ESBL-producing *Enterobacterales* predominating in high health status farms (environment and animals) and *Campylobacter* spp. in both high health status and low health status environments. Additionally, a high percentage of methicillin-resistant *Staphylococcus aureus* (MRSA) was found, mainly in environmental samples from high health status farms, and *Clostridioides difficile* was distributed ubiquitously among farms and samples. Furthermore, though less frequently, vancomycin-resistant *Enterococcus faecium* (VRE) was isolated only in high health status farms, and Gram-negative bacilli resistant to carbapenems were isolated only in environmental samples of high health status and low health status farms. This study underscores the importance of surveillance for MDR bacteria in farm animals and their environment, including their waste. Such ecosystems serve as crucial reservoirs of bacteria, requiring national-level surveillance to promote responsible antibiotic use and pandemic control.

## 1. Introduction

Antimicrobial resistance (AMR) is one of the largest threats to global health and food security [1]. The Organisation for Economic Cooperation and Development suggests that without the adoption of new policies, approximately 2.4 million individuals across North America, Australia and Europe could succumb to untreatable infections in the next three decades. Yet, the repercussions extend beyond human loss, encompassing an economic impact projected as exceeding USD 3.5 billion annually [1,2].

The primary contributor to AMR is antimicrobial use (AMU) [3], with its utilisation in human and veterinary medicine as the predominant factor [4]. Furthermore, AMU in veterinary medicine also plays an important role in the prevalence of AMR in human health [3]. Long-term AMU in food-producing animals fosters the emergence and dissemination of AMR through animals and environmental sources, since, for instance, a slurry is usually applied as a fertiliser for crops in agriculture. Notably, similarities between AMR bacteria found in humans and animals have been found in foodborne pathogens and commensal bacteria, including *Escherichia coli*, *Enterococcus* spp., or *Salmonella* [5]. 

The multidrug resistance (MDR) phenomenon diminishes treatment options in veterinary medicine as well as in human medicine, contributing to the spread of resistant bacteria through the food chain or by direct contact [6]. Traditionally, the swine sector has had a high level of antibiotic use in veterinary medicine [7,8]. As a consequence, MDR has relatively frequently been observed among microbial isolates from swine farms [9,10]. In addition, the post-weaning period is critical for piglets (21–28 days old), as they face stressors such as transport, diet changes, litter mixing and reduced maternal immunity, which can lead to susceptibility to Enterotoxigenic Escherichia coli (ETEC) and post-weaning diarrhea (PWD). Although antimicrobial treatments and management practices, such as biosecurity and diet adjustments, help control infectious diseases [11], some Spanish farms, despite standardising practices, experience frequent outbreaks and are classified as low health status (LHS). In contrast, others remain unaffected and are considered high health status (HHS). A farm with HHS must excel in production metrics and health records, with mortality rates under 4%, substandard pig percentages between 2–3% and treatment costs of EUR 1–1.5 per pig. HHS farms focus on selection and breeding and show superior biosecurity and resilience to major swine pathogens. In contrast, LHS farms experience recurrent post-weaning diarrhea and morbidity rates of 10–15%.

The three primary zoonotic bacteria in swine production are *Campylobacter*, *Salmonella* and livestock-associated methicillin-resistant *Staphylococcus aureus* (LA-MRSA) [12]. *Campylobacter* and *Salmonella* are also the most frequently reported foodborne zoonotic pathogens in human medicine in the European Union (EU), responsible for 61.3% and 29.2% of the foodborne infections reported in 2022, respectively [13]. In addition, zoonotic LA-MRSA can infect workers who have occupational contact with pigs [14]. Coagulase-negative *staphylococci*, such as *S. epidermidis*, *S. haemolyticus* and *S. saprophyticus*, are also being considered a major nosocomial burden, playing an important role as a reservoirs of AMR genes, which can be then transferred to *S. aureus* [6].

As antimicrobials are essential for the treatment of bacterial diseases [15], current policies focus on reducing AMU in livestock [16,17], particularly in the swine industry, which is the most extensive agricultural user of antimicrobials in the EU [18]. In this sense, the AMR surveillance of targeted zoonotic or bioindicator bacteria through European programmes [19] constitutes a fundamental pillar in the evaluation of the trends in AMR due to antimicrobial selection pressure [5,10]. 

Current policies have established strong control measures at farm level to minimise the administration of antibiotics, highlighting European [17] and national regulations, such as the National Action Plan against the Emergence of Antibiotic Resistance (PRAN) in Spain [20]. These plans are based on reducing the misuse of antibiotics in various sectors, including livestock farming. They focus on promoting responsible prescription and use of antibiotics, gathering data on AMU and collaborating across sectors to implement preventive measures to preserve the effectiveness of these drugs in human and animal health. 

However, to combat AMR, it is not only necessary to consider reducing AMU in animals but also to address the surrounding environment, which plays a key role as a potential reservoir for these MDR microorganisms [6]. The environment represents a stable microbial population among different animal batches and can sometimes host significant reservoirs of AMR [21]. Additionally, the prohibition of a large number of disinfectants by European authorities makes it increasingly difficult to combat these bacteria, especially those that are resistant and stable in the environment [6]. These stable communities exhibit characteristics specific to each type of production and, in some cases, characteristics unique to a particular company, production type or on-farm sanitary status [22]. 

Considering the aforementioned factors, understanding the epidemiology of AMR at the farm level represents a challenge crucial for the establishment of a robust field-level control programme. Consequently, the aim of this study was to assess the presence of multidrug-resistant (MDR) bacteria, enteric pathogens and resistance genes to the main antibiotics used in clinical practice both within the environment and in animals across pig farms characterized by varying degrees of sanitary status.

## 2. Results

### Detection of Multidrug Resistant Bacteria and Enteric Pathogens

After collecting 274 samples from 6 different pig farms (3 HHS and 3 LHS) and 2 different origins (environment and animals), a total of 9 groups of microorganisms were isolated. From farm environments, 36 samples were collected. On each farm, 10 samples from walls (2 pooled samples/farm), 10 from slats (2 pooled samples/farm) and 2 from slurry pit were collected. The groups most frequently detected were the ESBL-producing *Enterobacterales* (n = 210, 76.6 %), followed by *Campylobacter* spp. (n = 77, 28.1 %), MRSA (n = 32, 11.7 %), *C. difficile* (n = 22, 8 %), VRE (n = 5, 1.8 %), Gram-negative bacilli producing carbapenemases (n = 5, 1.8 %) and colistin-resistant *Enterobacterales* (n = 1, 0.4 %) (*p*-value < 0.05). Neither *Salmonella* nor *Yersinia* spp. were isolated in any sample.

**Figure 1:**
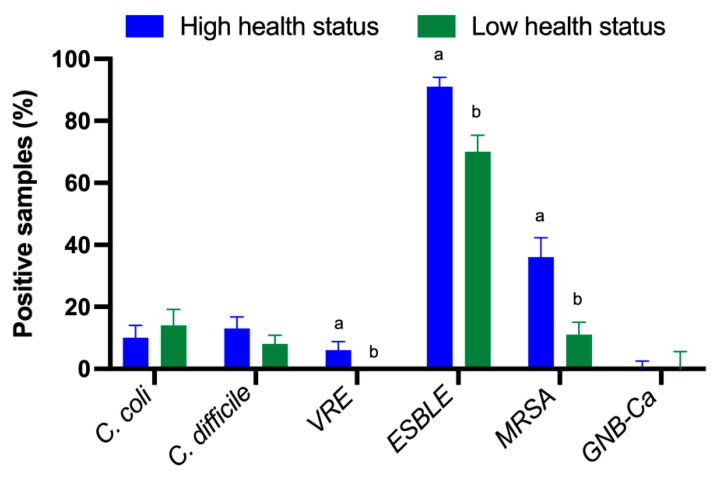
Percentage of each of the studied bacterial groups according to the farm status (High Health Status vs. Low Health Status). *C. coli*—*Campylobacter coli*. *C. difficile*—*Clostridioides difficile.* VRE—Vancomycin-resistant *Enterococcus*. ESBLE—ESBL-producing *Enterobacterales,* including *E. coli* and *K. pneumoniae*. MRSA—Methicillin-resistant *Staphylococcus aureus*. GNB-Ca—Gram-negative bacilli resistant to carbapenems. Different letters (a and b) represent significant differences within each bacterial group (*p*-value < 0.05). Data are presented as least squares means ± SE of the least squares means.

**Figure 2:**
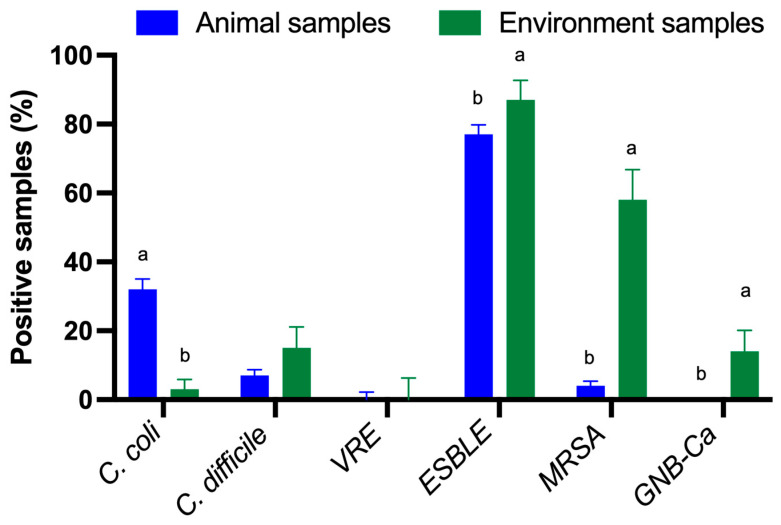
Percentage of each of the studied bacterial group according to the sample origin (environment vs. animals). *C. coli*—*Campylobacter coli*. *C. difficile*—*Clostridioides difficile*. VRE—Vancomycin-resistant *Enterococcus*. ESBLE—ESBL-producing *Enterobacterales,* including *E. coli* and *K. pneumoniae*. MRSA—Methicillin-resistant *Staphylococcus aureus*. GNB-Ca—Gram-negative bacilli resistant to carbapenems. Different letters (a and b) represent significant differences within each bacterial group (*p*-value < 0.05). Data are presented as least squares means ± SE of the least squares means.

Regarding the species of *Enterobacterales* studied, *E. coli* was identified in 98.6% of the positive samples and *Klebsiella pneumoniae* in 1.4%. ESBL-producing *Enterobacterales* significantly predominated in HHS farms compared to LHS farms (*p*-value < 0.001) (Figure 1). In addition, ESBLs were isolated from environmental and animal samples. However, no statistically significant differences were found between the presence of ESBL-producing *Enterobacterales* and the sample origin (environment vs. animals) (Figure 2). The data for each sample type related to the antimicrobial resistance (AMR) obtained, the sample origin and the status of the farm can be found in the Supplementary Materials.

All *Campylobacter* isolates were identified as *C. coli*. The percentage of *C. coli* isolated did not vary significantly between farm status (Figure 1). However, statistically significant differences were found between the percentage of the bacteria isolated and the origin of the sample (*p*-value < 0.001) (Figure 2).

MRSA was isolated at a higher percentage in HHS farms than in LHS farms (*p*-value < 0.001) (Figure 1), and it was also significantly detected colonising environmental samples compared to samples from animals (*p*-value < 0.001) (Figure 2). It should be noted that none of the MRSA strains isolated expressed the PBP2a protein.

The presence of *C. difficile* was evenly distributed both according to the farm status and the sample origin, with no significant differences found between any of the studied variables (Figure 1 and Figure 2). Only one strain of *C. difficile* tested positive for the presence of B toxin and negative for binary toxin.

In the case of VRE, all of them belonged to the species *Enterococcus faecium*, and they were only isolated in HHS farms (*p*-value = 0.032, Figure 1). Regardless of the presence of the bacteria and the origin of the sample, no statistically significant differences were found (Figure 2). None of the isolates expressed the vanA/B protein. 

In the case of Gram-negative bacilli resistant to carbapenemases, they were isolated at similar rates in both HHS and LHS farms. However, the bacteria were present only in environmental samples (*p*-value < 0.0001, Figure 2). The isolated strains belonged to the genus *Aeromonas* in 60% of cases and to the genus *Pseudomonas* in 40% of cases, with one of them expressing the carbapenemase OXA-48. 

Among all the samples studied, only one isolate of colistin-resistant *Enterobacterales* was detected, which was identified as *E. coli*, and the presence of the MCR-1 enzyme was not detected. This strain was isolated from an environmental sample obtained at one of the HHS farms.

## 3. Discussion

The results of this field study demonstrate that resistant zoonotic bacteria, such as ESBL-producing *Enterobacterales*, *C. coli*, MRSA, *C. difficile*, VRE and Gram-negative bacilli resistant to colistin or producing carbapenemases, are important microorganisms to control in pig production. The results obtained reveal that the presence of resistant zoonotic bacteria is not confined only to livestock, which are the primary focus of the main European programmes for controlling AMR. Instead, it highlights the critical importance of the farm environment in perpetuating these bacteria among consecutive batches of animals, posing a danger to both public and animal health.

*E. coli* is part of the intestinal microbiota in humans and animals, making it one of the most likely vectors for the dissemination of ESBLs [23]. Moreover, *E. coli* is the most frequent cause of human urinary tract infections and bloodstream infections, and it is one of the leading causative agents of foodborne infections worldwide [24]. Therefore, several institutions, such as the European Food Safety Authority (EFSA) and the European Centre for Disease Prevention and Control (ECDC), include ESBL-producing *E. coli* in their surveillance programmes [25,26]. Similarly, it is considered a candidate microorganism to assess the impact on policies for responsible antimicrobial use in the swine sector [6,25]. The data obtained in this study demonstrate the high prevalence of ESBL-producing *E. coli* in the pig farms studied, although none of them have used cephalosporins to treat the animals. Perhaps they are being co-selected by the use of other antimicrobials. These results support the importance of including ESBL *E. coli* screening in surveillance programmes. Additionally, it has been shown that strains isolated in animals can be transmitted to humans through direct contact or via the food chain, such as the *E. coli* lineage ST131, which is an extra-intestinal pathogen that can colonise the gastrointestinal tract of food-producing animals and humans [23]. In this line, the USA Center for Disease Control and Prevention reports showed a continuous increase in community-associated human infections caused by ESBL-producing *Enterobacterales*, and the high prevalence of infections by this bacterial group in humans has been attributed to the frequent use of third-generation cephalosporins in dairy farms [27]. 

*Campylobacter* and *Salmonella* are the two main enteric pathogens causing zoonoses in humans [12]. Pork is considered a major source of *Salmonella typhimurium* infection in humans in the EU, including monophasic strains (mST) and *Campylobacter* spp. [10,28,29]. Although our study did not identify any *Salmonella*, a widespread distribution of virulent serotypes, such as *S. typhimurium* and its monophasic variant, has emerged as a public health threat [10,28,29,30]. Despite the current situation, within the EU, there is no mandatory programme for the control of *Salmonella* at pork production level. On the other hand, our study shows that the swine sector constitutes an important reservoir of *C. coli*, but its clinical significance is unknown since most human infections are associated with the presence of *C. jejuni* [31,32], mainly related with chicken consumption [13]. It should be noted that the majority of *Campylobacter* spp. strains have been isolated from animal samples, but this does not indicate that they are not present in the environment, since *Campylobacter* spp., due to its physiological requirements, remains in the environment as a viable non-culturable form [33], which justifies its low recovery from environmental samples.

Regarding infection with *C. difficile*, an enteric pathogen also found on the studied farms, it is the leading cause of human nosocomial diarrhea, mainly associated with antibiotic use. Its occurrence in the community setting is becoming increasingly common [34]. This microorganism is also becoming a pathogen to watch out for in animal species, including pigs, horses and dairy calves [35,36,37]. It has been postulated that livestock could be one of the main reservoirs of *C. difficile* [34]. In line with this, studies have shown that both animals and farm workers can be colonised by identical clones of *C. difficile* [36]. This emphasises the need for additional research into the connection between this microorganism and the food chain. This study provides data demonstrating the significant prevalence of this pathogen on pig farms. In fact, there are studies reporting even higher rates of this pathogen in swine, with up to 87% of positive samples from pig farms [38,39,40,41]. Our work supports the importance of *C. difficile* as a pathogen to be considered within the One Health setting, as it has been isolated in all types of farms and samples.

Among Gram-positive cocci, the two main pathogens isolated therein were MRSA and VRE. Both are human pathogens, and studies have reported that patients infected with these resistant microorganisms present higher risk of mortality compared to those infected with non-resistant strains [42]. MRSA, frequently isolated in environmental samples from the studied farms, is an important pathogen causing infectious diseases in both humans and animals, leading to high economic costs in both public health and livestock. However, there is little information regarding the risk of transmission of these strains to humans, although the strains colonising animals are rarely isolated in humans [43]. Since the early 2000s, numerous studies have pointed to an increased risk of LA-MRSA colonisation among individuals who have prolonged, repeated contact with livestock, especially pigs [14]. The presence of this microorganism has been associated with factors including the administration of tetracycline and zinc oxide, the size and type of the pig herd and the management practices in place. Measures to control the spread of LA-MRSA on pig farms include conducting periodic tests for the detection of LA-MRSA in pigs and avoiding certain antibiotics [44,45].

Regarding human infections caused by VRE, they are highly prevalent in the USA and are gradually increasing in Europe. VRE poses a major healthcare problem due to the difficulty of treating serious conditions associated with this microorganism, given the significant therapeutic limitations available [46,47]. Traditionally, this difference is explained by the varying use of drugs from this family in different geographical areas. In addition to this phenomenon, one must consider the potential implication of using this microorganism as a probiotic, which may promote the emergence of these strains in the intestinal microbiome of animals [48]. These strains, after acquiring virulence factors, can be transmitted through direct contact between farmers, veterinarians and workers [49,50]. This risk becomes more serious considering that the dominant species of Enterococcus in pigs and humans are the same [51].

Finally, we have detected five isolates resistant to carbapenems, although only one of them was a carbapenemase producer, which is of major clinical interest. Thus, carbapenems have never been used in animal husbandry, and these isolates were detected in the environment of the farms, not in faecal samples. In particular, the OXA-48 isolate belonging to the genus *Aeromonas* is typically environmental. This finding is of concern, as horizontal transmission of resistance from environmental bacteria to commensal bacteria inhabiting the animal gut could result in pigs colonised with carbapenemase producing bacteria and posing a risk to consumers. Little is known about the influence of the food chain on the spread of carbapenem-resistant strains, although they have been isolated in pigs, cattle, poultry, seafood, pets and wildlife [52,53,54]. Due to the significant clinical importance of their spread, and although the frequency is very low, control measures should be strictly enforced to minimise their spread, especially in the environment through cleaning and disinfections procedures [55,56,57]. 

Regarding colistin resistance, it is important to note that it is one of the antibiotics used in the clinical management of so-called difficult-to-treat bacteria due to the scarcity of therapeutic alternatives. Therefore, the dissemination of strains resistant to this drug constitutes a significant healthcare problem. In fact, there are reports of emerging *E. coli* and *Klebsiella pneumoniae* strains resistant to this compound in human healthcare [58,59]. In our study, we detected the presence of a colistin-resistant *E. coli* that did not carry the MCR-1 plasmid. The presence of these types of proteins would add an additional component of severity due to the ease of transmission among other bacteria [60]. However, the high fitness cost associated with the acquisition and expression of this plasmid in *Enterobacterales* hinders the expansion of these strains [61]. The results obtained in this study demonstrate the excellent outcomes achieved in recent years in Spain following implementation of the “REDUCE-COLISTIN” programme led by the Spanish Agency of Medicines and Medical Products within the PRAN, which all Spanish swine companies have voluntarily joined [16].

The prevention and control of infections associated with MDR bacteria is a highly complex and challenging phenomenon. It is a multifactorial process involving diverse agents, such as the microbiome of all involved parties (patients, medical staff, healthcare workers, environment, etc.), ecosystem characteristics, dissemination pathways, clinical approaches and microbial resistome. The importance of the food chain is still poorly understood, especially in community-associated infections involving these types of bacteria [62]. To minimise this problem, the implementation of surveillance programmes that integrate data from humans (both nosocomial and community) and animals, especially those circulating in the food chain, are key. Currently, these surveillance programmes are multiple and scattered and should evolve with a One Health perspective [63]. Another aspect to consider is the improvement of antibiotic usage control in humans and animals. In fact, various, agencies such as the World Health Organisation (WHO) and the European Medicines Agency (EMA), have drawn up documents on the prudent use of antibiotics, both in humans and animals, as reported above [64]. 

Our study supports the need for surveillance for the presence of resistant zoonotic bacteria in both farm animals and their environment, including the waste generated. These results demonstrated that MDR bacteria were mainly found in environmental samples, suggesting that screening the environment is more sensitive than screening the animals. MDR bacteria from the environment provide a representation of the entire pool of animals coexisting in that space. In fact, different studies conducted solely with animal samples showed lower percentages of this type of microorganisms [65,66]. Furthermore, species typically exhibiting MDR profiles, such as clinically important ESBL-producing *Enterobacterales* or MRSA, are often physiologically adapted to harsh environmental conditions, being more difficult to eliminate from environmental reservoirs. Hence, it is necessary to introduce some of these microorganisms and resistance traits in national surveillance programmes through collaboration between governments and private companies in order to progress towards more sustainable farming with more responsible use of antibiotics, which will contribute to controlling this significant pandemic [32,57,67].

## 4. Materials and Methods

### 4.1. Study Sample

Over a four-month period, six houses from six pig farms were environmentally sampled. Farms were affiliated with one of the production companies that handle the majority of pigs slaughtered in Spain. The present study was approved by the Ethics Committee for Animal Experimentation from the Universitat Autònoma of Barcelona and the Animal Experimentation Commission from the local government (Departament d’Acció Climàtica, Alimentació i Agenda Rural from the Generalitat de Catalunya; Reference 12234).

### 4.2. Selection of High and Low Sanitary Status Farms to Define “Healthy” and “Unhealthy” Microbiomes

A farm attaining high health status (HHS) must meet stringent criteria. Firstly, it must demonstrate exceptional production performance, with metrics like average daily gain and feed conversion rate ranking in the top 25% of all pig farms. Secondly, it should maintain outstanding health records, placing among the top 10% for parameters such as mortality rate, substandard pig percentage during rearing and antimicrobial treatment costs. For these farms, expected mortality rates from weaning to slaughter should not exceed 4%, with substandard pig percentages between 2–3% and treatment costs ranging from EUR 1–1.5 per pig. Typically, such farms focus on selection and breeding. Conversely, low health status (LHS) farms are characterized by recurrent post-weaning diarrhea and morbidity rates of at least 10–15%. While standardized management practices are implemented across all farms, those with HHS exhibit superior performance in various aspects of biosecurity, including the acquisition of breeding pigs, visitor management, compartmental measures, equipment maintenance and sanitation practices. Additionally, HHS farms demonstrate resilience against major swine pathogens such as PRRSV, swine dysentery and Mycoplasma hyopneumoniae, with mortality rates below 4%.

### 4.3. Sampling

During January and June 2023, six pig farms were enrolled for in study (three HHS and three LHS). One house from each farm was selected according to the described criteria. At each farm, one house from the nursery facility containing sows with 3 week-old piglets was selected.

#### 4.3.1. Environmental Sampling

**Figure 3:**
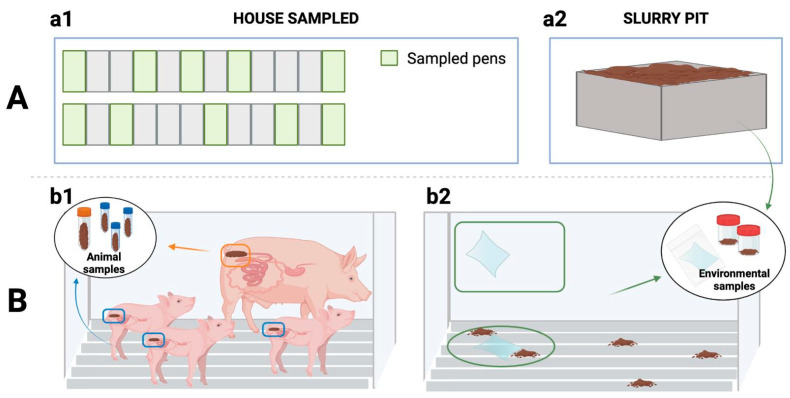
Sampling procedure. (**A**) Sampling points: (**a1**) Sampling within the house, (**a2**) Sampling outside the building (slurry pit). (**B**) Samples collected: (**b1**) from the animals (sows’ and piglets’ rectal faeces) and (**b2**) from the farm environment with swabs (wall and slat samples) and from slurry.

Thereafter, 10 pens within each house were sampled (walls and slats), i.e., 4 and 6 pens from the corners and the middle of the house, respectively (Figure 3). In addition, two slurry pit samples were collected from different sampling points in sterile pots (500 mL each). Wall and slat samples were collected by wiping 1 m^2^ of surfaces using both sides of a sterile wipe (Whirl-Pak^®^, Scharlab, Barcelona, Spain). First, wall samples (10 per farm, 1 per pen) were taken at an approximate height of 70 cm above the pen floor. Then, the slat samples (10 per farm, 1 per pen) were collected between and under the grates whenever possible. Once collected, all the samples were placed individually in a sterile bag with sterile diluent and transported to the laboratory in refrigerated conditions and processed within 12 h.

Once in the laboratory, the wall and slat samples were processed identically. Two pools of five wipes each (one quarter of a wipe per pool) were generated for each type of sample. The remaining wipes were stored in the freezer for further studies. Then, each pool and each slurry sample were placed in a stomacher (BagFilter^®^ 400 mL, Scharlab, Barcelona, Spain) and homogenised with 15 mL of PBS (5 min, 260 rpm).

#### 4.3.2. Animal Sampling

In each environmentally sampled pen, the sows and piglets were also sampled (Figure 3). Thus, faecal samples were collected directly from the rectum of sows (n = 10) and their piglets (n = 30, three piglets/sow). Each sample collected was homogenised, and a swab from each homogenised sample (Cary Blair sterile transport swabs, Deltalab^®^, Barcelona, Spain) was transported to the laboratory under refrigerated conditions and analysed within 24 h of collection.

### 4.4. Multidrug Resistant Bacteria and Enteric Pathogens Screening

When screening for MDR bacteria and enteric pathogens, a total of 274 samples were analysed (34 samples from the environment and 240 samples from the animals). The screening for MDR bacteria was carried out using selective plates for each microorganism. After this screening, the presence of the main antibiotic resistance mechanisms was analyzed in the phenotypically resistant strains through PCR and/or immunochromatography [68,69,70]

All the samples collected were cultured and analysed following the scientific protocols of the Spanish Society of Infectious Diseases and Clinical Microbiology (SEIMC) (https://seimc.org (accessed on 10 June 2024)) for detection of the bacterial groups related to antibiotic resistance: *Enterobacterales* resistant to third-generation cephalosporins (producing extended-spectrum beta-lactamases (ESBL)) and plasmid-mediated AmpC, Gram-negative bacilli resistant to carbapenems and/or colistin, MRSA, vancomycin-resistant *Enterococcus* (VRE) and enteric pathogens: *Clostridioides difficile*, *Salmonella*, *Yersinia* spp. and *Campylobacter* spp.

Isolation of ESBL-producing *Enterobacterales* was performed by seeding on a specific chromogenic medium, CHROMID ESBL (Biomerieux, Craponne, France), and selection of colistin-resistant strains was carried out by seeding on MacConkey Agar (Biomerieux, Craponne, France) combined with a colistin disc. For the detection of Gram-negative bacilli resistant to carbapenems, the chromogenic medium CROMID CARBA SMART (Biomerieux, Craponne, France) was used. Isolation of MRSA and VRE was performed using the CROMID MRSA SMART (Biomerieux, Crapone, France) and CHROMID VRE (Biomerieux, Crapone, France), respectively.

Regarding the detection of enteric pathogens, selective media were used for the isolation of *Campylobacter* spp. (Campylogel agar, Biomerieux, Crapone, France), *Yersinia* spp. (*Yersinia* CIN agar, Biomerieux, Crapone, France) and *C. difficile* (CHROMID *C. difficile* agar, Biomerieux, Crapone, France). In the case of *Salmonella,* prior to seeding on the chromogenic plate (BD Chromagar *Salmonella*, Becton, Dickinson, Franklin Lakes, NJ, USA), the sample underwent enrichment by broth (Selenite F broth, Biomerieux, Crapone, France) and subsequent incubation at 37 °C for 24 h.

All plates were incubated at 37 °C for 24 h, except for the selective plates for *Campylobacter*, which were maintained at 42 °C for 48 h under microaerophilic conditions, and the selective plates for *C. difficile*, which were maintained at 37 °C for 48 h under anaerobic conditions. From plates showing growth, the isolation of a colony was performed and the microorganism was subsequently identified using mass spectrometry (MALDI-TOF, Bruker, Karlsruhe, Germany).

Finally, rapid tests were conducted for confirmation and determination of resistance mechanisms in all the isolated phenotypically resistant microorganisms. Specifically, in MRSA, an immunochromatography test was performed to detect the PBP2a protein (Clearview PBP2a, Abbott, Lake County, IL, USA). Vancomycin resistance was confirmed by e-test (Biomerieux, Crapone, France) and detection of vanA/B genes was carried out using multiplex PCR (FilmArray multiplex PCR, Biomerieux, Crapone, France). ESBL-producing *Enterobacterales* were confirmed using a microdilution of cefotaxime and ceftazidime with and without clavulanic acid (MicroScan, Beckman Coulter, Indianapolis, IN, USA), and the presence of major carbapenemases (VIM, NDM, KPC, IMP and OXA-48) was confirmed using immunochromatography (NG-Test^®^ CARBA-5, NG-BIOTECH, Guipry-Messac, France). Colistin resistance was confirmed using microdilution with the UMIC colistin kit (Bruker, Karlsruhe, Germany), and the presence of the MCR 1 enzyme was determined using immunochromatography (NG-Test^®^ MCR-1, NG-BIOTECH, Guipry-Messac, France). Detection of genes encoding toxin B and binary toxin in *C. difficile* strains was performed using the Xpert C. difficile BT kit based on real-time PCR (GeneXpert, Cepheid, Sunnyvale, CA, USA).

### 4.5. Statistical Analysis

A generalised linear model (GLM), which assumed a binomial distribution for each AMR zoonotic bacterium presence, was fitted to the data to determine whether there was an association among the status of the farm (HHS and LHS), the sample origin (environment and animals) and the AMR zoonotic bacteria status of the batch. For this analysis, the error was designated as having a binomial distribution, and the probit link function was used. Binomial data for each sample were assigned: one if they had AMR zoonotic bacterium or zero if they did not. A *p*-value of less than 0.05 was considered statistically significant. Data are presented as least squares means ± standard error of the least squares means. All statistical analyses were carried out using a commercially available software program (SPSS 27.0.1.0; SPSS Inc., Chicago, IL, USA).

## 5. Conclusions

This study has identified *Campylobacter* and *C. difficile* among the enteric pathogens studied, while *Salmonella* or *Yersinia* were not isolated. Additionally, antimicrobial-resistant zoonotic bacteria of great importance in public and animal health were isolated, especially in the farm environment, suggesting the persistence of these microorganisms after cleaning and disinfection. Moreover, the presence of these microorganisms was particularly prevalent on those farms where the selective pressure of cleaning and disinfection was higher. Therefore, our results highlight the need for a holistic approach within the One Health paradigm, which acknowledges the interconnectedness between human, animal and environmental health in addressing zoonotic diseases and AMR.

## Data Availability

Data are contained within the article and Supplementary Materials.

## Supplementary Materials

The following supporting information can be downloaded at: https://www.mdpi.com/article/10.3390/antibiotics13090883/s1, Figure S1: Percentage of each of the studied bacterial groups according to the farm status (High Health Status vs. Low Health Status) and the animal sampled (sows or piglets). Figure S2: Percentage of each of the studied bacterial groups according to the farm status (High Health Status vs. Low Health Status) and the environmental sample collected (walls, slats or slurry pit).
