# Supplementary material for: Identification of Antimicrobial-Resistant Zoonotic Bacteria in Swine Production: Implications from the One Health Perspective
Source: Antibiotics (Basel). 2024 Sep 13;13(9):883. doi: 10.3390/antibiotics13090883 (PMC11428682; doi:10.3390/antibiotics13090883)
Supplement: Supplementary file 1 [file antibiotics-13-00883-s001.zip › antibiotics-3149926-supplementary.pdf]

antibiotics

Article

Identification of Antimicrobial-Resistant Zoonotic Bacteria in swine production: Implications from the One Health Perspective

Supplementary Material

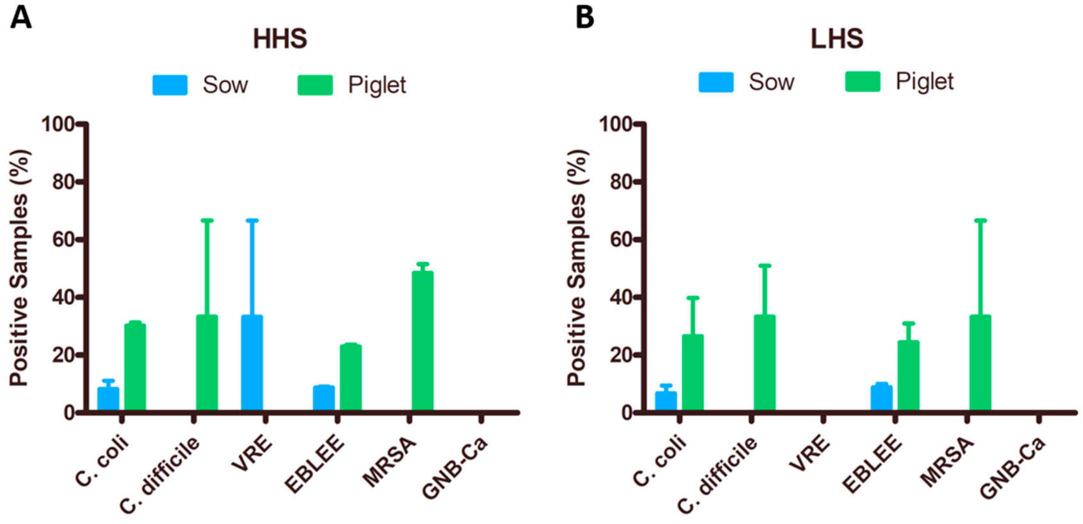

Figure S1: Percentage of each of the studied bacterial groups according to the farm status (High Health Status vs. Low Health Status) and the animal sampled (sows or piglets). A. Percentage of each studied bacterial group in High Health Status, based on the animal sampled (sows or piglets). B. Percentage of each studied bacterial group in Low Health Status, based on the animal sampled (sows or piglets). C. coli: *Campylobacter coli*. C. difficile: *Clostridioides difficile*. VRE: Vancomycin-resistant *Enterococcus*. ESBLE: ESBL-producing *Enterobacterales*, including *E. coli* and *K. pneumoniae*. MRSA: Methicillin-resistant *Staphylococcus aureus*. GNB-Ca: Gram-negative bacilli resistant to carbapenems. Data are presented as least squares means ± SE of the least squares means.

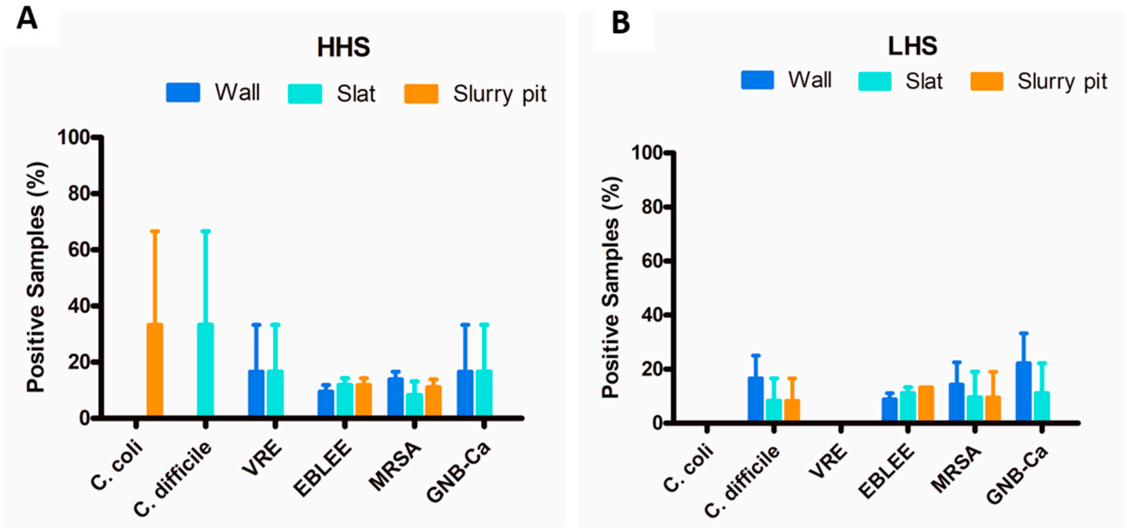

Figure S2: Percentage of each of the studied bacterial groups according to the farm status (High Health Status vs. Low Health Status) and the environmental sample collected (walls, slats or slurry pit). A. Percentage of each studied bacterial group in High Health Status, based on the type of sample collected. B. Percentage of each studied bacterial group in Low Health Status, based on the type of sample collected. C. coli: *Campylobacter coli*. C. difficile*: Clostridioides difficile.* VRE: Vancomycin-resistant *Enterococcus.* ESBLE: ESBL-producing *Enterobacterales*, including *E. coli* and *K. pneumoniae*. MRSA: Methicillin-resistant *Staphylococcus aureus*. GNB-Ca: Gram-negative bacilli resistant to carbapenems. Data are presented as least squares means ± SE of the least squares means.
